# Reliability of imaging assessment of fatty infiltration in rotator cuff disorders: protocol for a systematic review and meta-analysis

**DOI:** 10.1136/bmjopen-2026-120564

**Published:** 2026-08-11

**Authors:** Jinrong Lin, Zhenming Zhang, Shurong Zhang, Xiaoyu Tang, Cheng Chen, Hongyu Bai, Siyang Liu, Jeremy S Lewis, Jean-Sebastien Roy, Kat Steiner, Xin Ma, Shiyi Chen, Jiwu Chen, Jonathan Rees, Sally Hopewell

**Affiliations:** 1 Nuffield Department of Orthopaedics, Rheumatology and Musculoskeletal Sciences, Botnar Institute for Musculoskeletal Sciences, University of Oxford, Oxford, UK; 2 Shanghai Sixth People’s Hospital, National Centre for Orthopaedics, Shanghai Jiao Tong University School of Medicine, Shanghai, China; 3 Huashan Hospital, Department of Sports Medicine, Fudan University, Shanghai, China; 4 Academy of Pharmacy, Xi’an Jiaotong-Liverpool University, Suzhou, China; 5 Shanghai General Hospital, Department of Sports Medicine, Shanghai Jiao Tong University, Shanghai, China; 6 Huashan Hospital, Department of Orthopaedics, Fudan University, Shanghai, China; 7 Central London Community Healthcare National Health Service Trust, London, UK; 8 School of Health and Social Work, University of Hertfordshire, Hatfield, UK; 9 School of Life and Health Sciences, University of Nicosia, Nicosia, Cyprus; 10 Clinical Therapies, University of Limerick, Limerick, Ireland; 11 School of Rehabilitation Sciences, Université Laval Faculté de médecine, Quebec City, Quebec, Canada; 12 Centre for Interdisciplinary Research in Réactualisation and Social Integration, CIUSSS de la Capitale-Nationale, Québec City, Quebec, Canada; 13 Bodleian Health Care Libraries, University of Oxford, Oxford, UK; 14 Centre for Statistics in Medicine, University of Oxford Nuffield Department of Orthopaedics Rheumatology and Musculoskeletal Sciences, Oxford, UK

**Keywords:** SPORTS MEDICINE, Shoulder, Musculoskeletal disorders, Diagnostic Imaging, Meta-Analysis, Systematic Review

## Abstract

**Abstract:**

**Introduction:**

Fatty infiltration (FI) of the rotator cuff (RC) muscles reflects chronic deterioration in muscle quality associated with tendon injury, disuse, ageing and nerve injury. Accurate and reliable assessment of FI based on imaging interpretation is essential for evaluating muscle degeneration, guiding rehabilitation planning and enabling meaningful comparisons across clinical and research settings. Several semi-qualitative grading systems and quantitative measurement approaches across imaging modalities, including CT, MRI, ultrasound and advanced techniques such as Dixon MRI have been used to assess FI; however, the reproducibility of FI grading or quantification at the level of image interpretation varies widely. This protocol outlines a systematic review and meta-analysis designed to evaluate the inter-rater and intra-rater reliability of imaging-based FI assessment at the level of image interpretation in RC disorders.

**Methods and analysis:**

The review will follow the Preferred Reporting Items for Systematic Review and Meta-Analyses Protocols (PRISMA-P) guidelines and is preregistered in PROSPERO (CRD420251242564). Searches will be conducted in MEDLINE, Embase, CINAHL, Web of Science and the Cochrane Library from inception to the search date. Two reviewers will independently screen, extract data and assess study quality using the Quality Appraisal of Reliability Studies (QAREL) checklist. The COnsensus-based Standards for the selection of health Measurement INstruments (COSMIN) framework will be used to guide interpretation of reliability-related measurement properties. The primary outcomes will be inter-rater and intra-rater reliability reflecting the consistency of image-based FI grading or quantification. Where appropriate, pooled reliability estimates will be calculated separately by imaging modality (eg, MRI, CT and US) and by type of reliability (inter-rater and intra-rater). When quantitative pooling is not feasible because of limited studies or substantial heterogeneity, findings will be synthesised narratively and presented in summary tables. These will be synthesised using a restricted maximum likelihood random-effects model in R software, version 4.3.2 (R Foundation for Statistical Computing, Vienna, Austria) with the metafor package.

**Ethics and dissemination:**

Ethical approval is not required as the review will use only published data. The results will be disseminated through a peer-reviewed publication and conference presentations to inform research and clinical practice in musculoskeletal imaging.

**PROSPERO registration number:**

CRD420251242564.

**STRENGTHS AND LIMITATIONS OF THIS STUDY**

- This preregistered protocol follows the Preferred Reporting Items for Systematic Review and Meta-Analyses Protocols (PRISMA-P) guidelines and the COnsensus-based Standards for the selection of health Measurement INstruments (COSMIN) framework to ensure transparent and methodologically robust evaluation of measurement properties.
- The review applies a structured and standardised approach to evaluate inter-rater and intra-rater reliability using the Quality Appraisal of Reliability Studies (QAREL) checklist for risk-of-bias appraisal and the COSMIN framework to inform interpretation of reliability evidence.
- A comprehensive search strategy will include five major databases and dual independent review at all stages to minimise selection and extraction bias.
- Planned subgroup analyses will explore potential sources of heterogeneity related to imaging methods, raters and pathology characteristics.
- Limitations include potential heterogeneity in imaging parameters, rater experience and study design, which may influence pooled estimates despite planned subgroup and sensitivity analyses.

## Introduction

Shoulder pain is a common musculoskeletal complaint in active athletic and non-athletic populations.^1^ A large proportion of cases involve rotator cuff (RC) disorders, a multifactorial condition that can affect tendon, muscle and related structures such as bursal tissue. It may progress along a degenerative continuum.^2 3^ One of the key pathological features is fatty infiltration (FI), defined as the replacement of contractile fibres by adipose tissue within RC muscles,^4^ reflecting chronic deterioration in muscle quality associated with tendon injury, disuse, ageing and nerve injury.^3 5^ In sports and rehabilitation settings, it is increasingly recognised as an indicator of muscle health, influencing shoulder strength, movement control and recovery potential.^6^ FI is also incorporated into clinical decision-making, including judgements about reparability, prognosis and expected rehabilitation potential, which inherently assumes that FI assessment is reproducible when interpreted by clinicians and researchers.

Imaging is essential for quantifying FI and tracking changes in muscle composition. Several radiological methods have been developed, yet no single approach is universally accepted.^7^ The Goutallier classification, first described using CT and later adapted for MRI, remains widely used but is subjective and two-dimensional visual grading.^7^ Ultrasound enables static and dynamic evaluation of tissues, but is influenced by equipment and user technical skill,^8^ while advanced MRI techniques such as Dixon imaging and MR spectroscopy (MRS) provide quantitative estimates of intramuscular fat.^9^ Despite these advances, reported inter-rater and intra-rater reliability across modalities ranges from poor to almost perfect agreement.^8 10–12^ Differences in imaging acquisition parameters, sequence selection, anatomical level of assessment, rater training and professional background, centre-level variation and the semi-qualitative grading systems or quantitative measurement approaches used are all likely contributors to the substantial variability in how consistently FI is interpreted from imaging.

In imaging-based FI assessment, reliability reflects how consistently FI is interpreted by different raters or by the same rater on repeated interpretation of the same images. Recent reviews have evaluated FI as a prognostic factor noting substantial heterogeneity in how FI is measured across imaging modalities.^13 14^ However, none has specifically examined the reliability of these measurement approaches. This unresolved uncertainty regarding interpretation-level reliability limits the interpretability of outcome studies and prevents standardisation of FI assessment in clinical and research settings,^15^ highlighting the need for a clear synthesis of reliability evidence.

This protocol outlines a systematic review and meta-analysis to identify, appraise and synthesise evidence on the inter-rater and intra-rater reliability of imaging-based FI assessment in RC disorders. The review will compare semi-qualitative grading systems and quantitative measurement approaches across modalities and explore factors influencing inter-rater and intra-rater reproducibility at the level of image interpretation. The findings will support standardisation of FI assessment and guide its use in research, rehabilitation and clinical decision-making.

## Methods and analysis

This protocol was developed in accordance with the Preferred Reporting Items for Systematic Review and Meta-Analyses Protocols (PRISMA-P) guidelines^16^ to ensure transparent reporting. It has been preregistered in the PROSPERO international database (registration number: CRD420251242564). Any important amendments to this protocol will be documented in PROSPERO and reported in the final systematic review.

### Eligibility criteria

Studies will be selected based on predefined criteria for the study population, imaging method, outcomes and study design. A summary of inclusion and exclusion criteria is presented in table 1.

**Table 1:** Eligibility criteria

Criteria	Inclusion criteria	Exclusion criteria
Population	Studies involving human participants from either healthy or clinical populations who have undergone imaging, with any degree of RC tendinopathy, partial-thickness or full-thickness RC tear identified	Studies involving non-human, cadaveric or animal samples
Instruments	Imaging methods used to assess FI of RC muscles based on image interpretation, including CT, CTA, MRI, MRA, Dixon imaging, MRS or ultrasound; both semi-qualitative (eg, Goutallier classification) and quantitative (eg, fat fraction and echo intensity) approaches will be included; no restrictions will be placed on the specific quantification protocol used	Studies not using imaging methods to assess FI or focusing solely on other structural parameters (eg, tendon integrity and muscle volume) without FI quantification
Comparators	Not applicable. This review evaluates the reliability of FI assessment methods, not comparisons between interventions	Not applicable
Outcomes	Studies reporting intra-rater and/or inter-rater reliability reflecting consistency of image-based FI grading or quantification, using established statistics (eg, κ or ICC)	Studies without quantitative reliability data or lacking sufficient detail to extract reliability statistics.
Study design	Any original study design reporting measurement reliability of FI assessment; both primary and secondary reliability analyses are eligible	Review papers, conference abstracts, case reports, editorials or methodological notes without empirical reliability data
Language	No language restrictions; where necessary, translation will be undertaken using appropriate tools or support	Not applicable
Publication date	No restriction by publication year (from inception to search date)	Not applicable

CTA, CT arthrography; FI, fatty infiltration; ICC, intraclass correlation coefficient; MRA, magnetic resonance arthrography; MRS, magnetic resonance spectroscopy; RC, rotator cuff; κ, weighted Cohen’s kappa.

### Information sources

A comprehensive electronic search will be conducted across multiple databases from their inception to the search date, including MEDLINE, Embase, CINAHL, Web of Science and the Cochrane Library. Reference lists of eligible studies and related reviews will also be screened. No date restrictions will be applied. No language restrictions. Where necessary, translation will be undertaken using appropriate tools or support.

### Search strategy

Full search strategies for all databases are provided in the online supplemental material 1. The search strategy was designed by KS, a medical librarian, and will be translated appropriately for each database. The search strategy includes free-text keywords and subject headings, where available. Key concepts are (1) RC, including specific muscle names; (2) FI, fatty degeneration, Goutallier and similar terms; (3) terms relating to reliability, validity, reproducibility, inter-observer and intra-observer, and kappa coefficient; and (4) diagnostic imaging, specifically MRI, CT and ultrasound. The search terms are combined as (1 AND 2 AND 3) OR (1 AND 2 AND 4) – this is to provide the most comprehensive search strategy to find papers which may not mention validity or reliability of diagnostic imaging in the title or abstract of the paper, but only in the full text. Filters to remove animal studies and excluded study types will be applied, where available, and no other limits or filters will be used. Results will be deduplicated using Covidence (Veritas Health Innovation, Melbourne, Australia).

### Study records

#### Data management

All retrieved records will be imported into Covidence for automatic deduplication and blinded screening. Each record will be independently screened by two reviewers according to the eligibility criteria. Two reviewers will independently screen all titles and abstracts, retrieve potentially relevant full texts and assess eligibility. A standardised Excel form will be developed to extract study details, imaging methods, rater characteristics and reliability statistics (eg, weighted Cohen’s κ or intraclass correlation coefficient (ICC)). The form will be pilot-tested and refined as required, with all modifications documented in the final report. Only published data will be analysed; where necessary, attempts to contact study authors on two occasions to obtain missing or unclear information will be facilitated by the lead author.

### Selection process

Titles and abstracts of all retrieved records will be screened independently by two reviewers against the eligibility criteria summarised in table 1. Full texts of potentially eligible studies will then be assessed to determine final inclusion. Disagreements will be resolved by discussion or, if necessary, consultation with a third reviewer. All stages will be documented in a PRISMA^17^ flow diagram.

### Review process and disagreement resolution

Two reviewers with clinical and research experience in musculoskeletal and sports medicine, and with prior training in systematic review methodology, will independently perform all key stages of the review, including study selection, data extraction and quality assessment. A pilot screening of several studies will be conducted at the outset to ensure consistency in applying the eligibility criteria. Any discrepancies between reviewers will first be discussed to reach consensus. If disagreement persists, a third reviewer with expertise in musculoskeletal imaging of the shoulder will adjudicate the final decision. All decisions and justifications for exclusion will be documented for transparency.

### Data items

The following information will be extracted from each included study using a standardised Excel form:

1. Study characteristics: first author, publication year, country, study design, recruitment setting and sample size (total and per analysis where applicable).
2. Population details: participant demographics (age, sex), clinical or symptomatic status (eg, asymptomatic, shoulder pain, RC tendinopathy, partial-thickness or full-thickness tear, tear size or severity where available), inclusion and exclusion criteria and recruitment methods.
3. Imaging and assessment methods: imaging modality (CT, CTA, MRI, MRA, Dixon imaging, MRS or ultrasound), technical and sequence details (eg, T1-weighted, T2-weighted and Dixon echo numbers), assessment plane, muscles examined (supraspinatus, infraspinatus, subscapularis and teres minor) and classification or quantification method used (eg, Goutallier grade, fat fraction and echo intensity). For quantitative approaches, details of image acquisition, processing methods and standardisation procedures will also be extracted where available.
4. Rater and methodological details: number and background of raters, level of experience or training, blinding procedures, order and independence of assessment, time interval between repeated measures (for intra-rater reliability) and other information required for quality appraisal using the Quality Appraisal of Reliability Studies (QAREL) checklist.^18^
5. Reliability outcomes: type of reliability assessed (intra-rater and/or inter-rater), statistical coefficient reported (eg, ICC or κ), specification of the statistical model where applicable (eg, ICC model, single or average measures, consistency or absolute agreement), point estimates with corresponding measures of precision (eg, 95% CIs or SEs).

### Risk of bias in individual studies

The methodological quality will be assessed using the QAREL checklist.^18^ The QAREL tool consists of 11 items that evaluate potential sources of bias in reliability studies, including representativeness of participants and raters, blinding procedures, order effects, time intervals between repeated measures and appropriateness of statistical analyses. Each item will be rated as ‘yes’, ‘no’, ‘unclear’, or ‘not applicable’, based on operational definitions adapted for FI assessment (online supplemental material 2). Two reviewers will independently perform the quality assessment, with discrepancies resolved by discussion or consultation with a third reviewer. A study will be considered of high quality if at least half of applicable items receive a ‘yes’ rating. Results will be summarised descriptively and presented in tables.

### Data synthesis

A narrative synthesis will describe inter-rater and intra-rater reliability of FI assessment across imaging modalities and methods.

Where studies are sufficiently comparable in design, population and outcomes, meta-analysis will be performed. Separate meta-analyses will be conducted for each type of reliability coefficient (eg, weighted Cohen’s κ or ICC), and stratified by type of reliability (inter-rater and intra-rater) and imaging modality. ICC estimates will be transformed using Fisher’s z transformation prior to pooling and back-transformed for presentation. Pooled reliability estimates will be calculated in R (V.4.3.2; R Foundation for Statistical Computing, Vienna, Austria) using the metafor package, with a restricted maximum likelihood random-effects model.^19^

Subgroup meta-analyses will be conducted where data permit, according to assessment method (semi-qualitative and quantitative), muscle (supraspinatus, infraspinatus, subscapularis and teres minor), rater profession, imaging plane and type of RC pathology (eg, tendinopathy, partial-thickness tear and full-thickness tear). Meta-analysis will not be performed for subgroups including fewer than three studies; instead, findings from these subgroups will be synthesised narratively and presented in summary tables.

Heterogeneity will be assessed using Cochran’s Q test and quantified using the *I*² statistic, and will be visualised using forest plots. Publication bias will be assessed by funnel plots and Egger’s regression test where at least 10 studies are available; the trim-and-fill method will be applied if asymmetry is detected. If quantitative pooling is not feasible, findings will be presented narratively, supported by summary tables that describe reliability estimates, study characteristics and quality ratings.

The COnsensus-based Standards for the selection of health Measurement INstruments (COSMIN) framework will be used to guide interpretation of the overall quality of reliability evidence across studies.

### Patient and public involvement

No patients or members of the public were involved in the design, conduct or reporting of this systematic review protocol. This review focuses on the methodological evaluation of imaging reliability studies and does not involve direct patient data or participation.

## Ethics and dissemination

This study does not require formal ethical approval as it will use only previously published and publicly available data, with no involvement of human participants or collection of primary data. The findings will be disseminated through publication in a peer-reviewed journal and presentation at academic and clinical conferences to inform future research and practice in musculoskeletal imaging.

## Discussion

FI of the RC muscles reflects enduring muscle quality changes associated with tendon injury, disuse, ageing and nerve injury. In athletic and non-athletic individuals, advanced FI has been linked to impaired shoulder strength, altered biomechanics and poorer recovery after injury. Reliable assessment of FI is therefore essential for evaluating muscle function, guiding rehabilitation and monitoring response to both conservative and surgical interventions. Despite this importance, substantial variability exists in how FI is quantified across imaging modalities and assessment protocols.

This systematic review and meta-analysis will synthesise current evidence on the intra-rater and inter-rater reliability of imaging-based FI measurements in RC muscles. By adhering to the PRISMA-P and QAREL methodological standards, the review will compare semi-qualitative grading systems and quantitative measurement approaches to identify factors influencing reliability across different techniques, muscles and rater groups. The findings will help establish more standardised, reproducible imaging methods for assessing muscle quality in shoulder injury research and clinical practice.

Several limitations should be considered. Variability in imaging parameters, FI grading systems and rater experience may contribute to residual heterogeneity. In addition, differences in statistical methods used to assess reliability (eg, ICC models and kappa statistics) may limit comparability and preclude quantitative synthesis in some cases. The overall strength of the evidence may also be constrained by the methodological quality of the included studies, and publication bias cannot be excluded. Nevertheless, by systematically evaluating methodological quality using the QAREL framework and integrating data across diverse imaging approaches, this study aims to provide an evidence-based reference for selecting reliable and practical FI assessment methods in both sports medicine and general musculoskeletal care.

## Supplementary material

10.1136/bmjopen-2026-120564 online supplemental file 1

10.1136/bmjopen-2026-120564 online supplemental file 2

## Data Availability

Data sharing not applicable as no datasets generated and/or analysed for this study.
