# Supplementary material for: Reliability of imaging assessment of fatty infiltration in rotator cuff disorders: protocol for a systematic review and meta-analysis
Source: BMJ Open. 2026 Aug 11;16(8):e120564. doi: 10.1136/bmjopen-2026-120564 (PMC13475469; doi:10.1136/bmjopen-2026-120564)
Supplement: Supplementary file 1 [file bmjopen-2026-120564.reviewer_comments.pdf]

PEER REVIEW HISTORY

BMJ Open publishes all reviews undertaken for accepted manuscripts. Reviewers are asked to complete a checklist review form and are provided with free text boxes to elaborate on their assessment. These free text comments are reproduced below.

ARTICLE DETAILS

Title (Provisional)

Reliability of imaging assessment of fatty infiltration in rotator cuff disorders: a protocol for a systematic review and meta-analysis

Authors

Lin, Jinrong; Zhang, Zhenming; Zhang, Shurong; Tang, Xiaoyu; Chen, Cheng; Bai, Hongyu; Liu, Siyang; Lewis, Jeremy S; Roy, Jean-Sebastien; Steiner, Kat; Ma, Xin; Chen, Shiyi; Chen, Jiwu; Rees, Jonathan; Hopewell, Sally

---

VERSION 1 - REVIEW

**Reviewer**	**1**
**Name**	**Wei, Shijun**
**Affiliation**	**Wuhan General Hospital of Guangzhou Command, Orthopaedics**
**Date**	**24-May-2026**
**COI**	**None**

Thank you for the opportunity to review this well-written protocol. The manuscript is suitable for publication after minor revisions. Below are questions/clarifications for the authors to address.

1. **Handling of subgroups with fewer than three studies**: You mention that meta-analysis will not be performed for subgroups with fewer than three studies. Could you specify how these subgroups will be synthesised narratively—e.g., will you present them in tables with descriptive summaries?
2. **Clarity on “quantitative measurement approaches”**: In the introduction and methods, you include quantitative approaches such as fat fraction (MRI) and echo intensity (US). For US, echo intensity is often expressed in decibels or grayscale units, but there is no universally standardised method. Will you require studies to have used a validated quantitative protocol (e.g., histogram analysis with standardised machine settings), or will any attempt at numeric quantification be included? Please specify.

3. Lack of clarity on handling situations where meta-analysis is not feasible: The abstract notes that pooled estimates will be calculated "where appropriate". It does not specify what will be done when meta-analysis is not possible (e.g., fewer than three studies per subgroup or excessive heterogeneity). The full protocol describes narrative synthesis with summary tables in such cases. Please amend the abstract to briefly mention this alternative approach.

VERSION 1 - AUTHOR RESPONSE

REVIEWER COMMENTS

Comment 1

Handling of subgroups with fewer than three studies: You mention that meta-analysis will not be performed for subgroups with fewer than three studies. Could you specify how these subgroups will be synthesised narratively—e.g., will you present them in tables with descriptive summaries?

Response

We have clarified how subgroups with fewer than three studies will be handled in the Data Synthesis section.

Changes made: Page 9.

"Meta-analysis will not be performed for subgroups including fewer than three studies; instead, findings from these subgroups will be synthesised narratively and presented in summary tables."

Comment 2

Clarity on "quantitative measurement approaches": In the introduction and methods, you include quantitative approaches such as fat fraction (MRI) and echo intensity (US). For US, echo intensity is often expressed in decibels or grayscale units, but there is no universally standardised method. Will you require studies to have used a validated quantitative protocol (e.g., histogram analysis with standardised machine settings), or will any attempt at numeric quantification be included? Please specify.

Response

We do not intend to restrict eligibility based on the use of a specific validated quantitative protocol. Restricting inclusion to studies using predefined validated protocols could exclude relevant reliability studies and may be difficult to apply consistently given the lack of universally accepted standards for some quantitative approaches, particularly ultrasound echo intensity. To clarify this, we have revised the eligibility criteria to state that quantitative

approaches will be eligible if they provide a numerical estimate of fatty infiltration. Methodological details related to image acquisition, processing, and standardisation procedures will be extracted and considered when interpreting reliability estimates and potential sources of heterogeneity.

Changes made: Page 5; Page 8.

“No restrictions will be placed on the specific quantification protocol used.”

“For quantitative approaches, details of image acquisition, processing methods, and standardisation procedures will also be extracted where available.”

Comment 3

Lack of clarity on handling situations where meta-analysis is not feasible: The abstract notes that pooled estimates will be calculated “where appropriate”. It does not specify what will be done when meta-analysis is not possible (e.g., fewer than three studies per subgroup or excessive heterogeneity). The full protocol describes narrative synthesis with summary tables in such cases. Please amend the abstract to briefly mention this alternative approach.

Response

We agree that this should be stated more clearly in the abstract.

Changes made: Page 2.

“When quantitative pooling is not feasible because of limited studies or substantial heterogeneity, findings will be synthesised narratively and presented in summary tables.”

Additional revisions

We also corrected a minor inconsistency in the “Strengths and limitations of this study” section, where “six major databases” has been revised to “five major databases” to reflect the databases included in the search strategy.

We thank the Editor and Reviewer for their consideration of our manuscript.
