# Supplementary material for: Reliability of imaging assessment of fatty infiltration in rotator cuff disorders: protocol for a systematic review and meta-analysis
Source: BMJ Open. 2026 Aug 11;16(8):e120564. doi: 10.1136/bmjopen-2026-120564 (PMC13475469; doi:10.1136/bmjopen-2026-120564)
Supplement: Supplementary file 2 [file bmjopen-16-8-s001.docx]

**Appendix 1 Search strategies**

**1. MEDLINE (Ovid interface)**

| Run | | 27 March 2026 |  |  |
| --- | --- | --- | --- | --- |
|  |  | | |  |
| 1 | Rotator Cuff/ | | | 9,447 |
| 2 | Rotator Cuff Injuries/ | | | 9,231 |
| 3 | rotator cuff*.tw,kf. | | | 19,509 |
| 4 | (supraspinat* or supra-spinat* or infraspinat* or infra-spinat* or subscapular* or sub-scapular* or teres minor*).tw,kf. | | | 12,390 |
| 5 | 1 or 2 or 3 or 4 | | | 28,054 |
| 6 | (fatty infiltration or fatty degeneration or intramuscular fat or intra-muscular fat or muscular fat or fat fraction or muscle fat or Goutallier).tw,kf. | | | 14,378 |
| 7 | exp "Reproducibility of Results"/ | | | 510,963 |
| 8 | Observer Variation/ | | | 46,962 |
| 9 | Validation Study/ | | | 124,944 |
| 10 | (reliability or reliable or reproducibility or reproducible or repeatability or repeatable or agreement or interobserver or intraobserver or inter-observer or intra-observer or interrater or intrarater or inter-rater or intra-rater or kappa or intraclass correlation or intra-class correlation or validat* or validity).tw,kf. | | | 2,289,484 |
| 11 | 7 or 8 or 9 or 10 | | | 2,523,205 |
| 12 | (imag* or (computed adj3 tomograph*) or CT or CTs or magnetic resonance imag* or MRI or MRIs or ultraso*).tw,kf. | | | 2,794,451 |
| 13 | exp Magnetic Resonance Imaging/ or exp Tomography, X-Ray Computed/ or exp Ultrasonography/ or Diagnostic Imaging/ | | | 1,534,033 |
| 14 | 12 or 13 | | | 3,302,325 |
| 15 | 5 and 6 and 11 | | | 232 |
| 16 | 5 and 6 and 14 | | | 934 |
| 17 | 15 or 16 | | | 972 |
| 18 | exp Animals/ not Humans/ | | | 5,439,535 |
| 19 | 17 not 18 | | | 925 |
| 20 | case report*.ti. | | | 414,772 |
| 21 | 19 not 20 | | | 916 |
| 22 | limit 21 to (case reports or editorial or meta analysis or network meta-analysis or "review" or "scientific integrity review" or "scoping review" or "systematic review") | | | 72 |
| 23 | 21 not 22 | | | 844 |

**2. Embase (Ovid interface)**

| Run | | 27 March 2026 |  |  |
| --- | --- | --- | --- | --- |
|  | |  |  |  |
| 1 | exp rotator cuff/ | | | 14,717 |
| 2 | exp rotator cuff injury/ | | | 20,613 |
| 3 | rotator cuff*.tw,kf. | | | 24,372 |
| 4 | (supraspinat* or supra-spinat* or infraspinat* or infra-spinat* or subscapular* or sub-scapular* or teres minor*).tw,kf. | | | 15,839 |
| 5 | 1 or 2 or 4 | | | 37,424 |
| 6 | (fatty infiltration or fatty degeneration or intramuscular fat or intra-muscular fat or muscular fat or fat fraction or muscle fat or Goutallier).tw,kf. | | | 19,575 |
| 7 | 5 and 6 | | | 1,753 |
| 8 | reproducibility/ | | | 300,357 |
| 9 | observer variation/ | | | 23,551 |
| 10 | exp reliability/ | | | 309,403 |
| 11 | exp validity/ | | | 167,659 |
| 12 | exp measurement repeatability/ | | | 43,039 |
| 13 | validation study/ | | | 130,926 |
| 14 | kappa statistics/ | | | 13,153 |
| 15 | (reliability or reliable or reproducibility or reproducible or repeatability or repeatable or agreement or interobserver or intraobserver or inter-observer or intra-observer or interrater or intrarater or inter-rater or intra-rater or kappa or intraclass correlation or intra-class correlation or validat* or validity).tw,kf. | | | 3,032,887 |
| 16 | 8 or 9 or 10 or 11 or 12 or 13 or 14 or 15 | | | 3,227,824 |
| 17 | (imag* or (computed adj3 tomograph*) or CT or CTs or magnetic resonance imag* or MRI or MRIs or ultraso*).tw,kf. | | | 4,111,598 |
| 18 | exp nuclear magnetic resonance imaging/ or exp computer assisted tomography/ or exp exp echography/ or diagnostic imaging/ | | | 2,985,110 |
| 19 | 17 or 18 | | | 5,112,869 |
| 20 | 7 and 16 | | | 301 |
| 21 | 7 and 19 | | | 1,237 |
| 22 | 20 or 21 | | | 1,281 |
| 23 | (exp animal/ or nonhuman/) not exp human/ | | | 7,969,280 |
| 24 | 22 not 23 | | | 1,201 |
| 25 | case report*.ti. | | | 494,899 |
| 26 | 24 not 25 | | | 1,189 |
| 27 | limit 26 to ("clinical trial (clinicaltrials.gov)" or conference abstract or editorial or "review") | | | 337 |
| 28 | 26 not 27 | | | 852 |

**3. CINAHL (EBSCOhost)**

| Run | | 27 March 2026 |  |  |
| --- | --- | --- | --- | --- |
| Limits | | Proximity; Apply equivalent subjects |  |  |
| S28 | S26 NOT S27 | | | 348 |
| S27 | PT "review" OR TI "review" | | | 680,069 |
| S26 | S24 NOT S25 | | | 363 |
| S25 | (MH "Invertebrates+" OR MH "Vertebrates+") NOT (MH "Human") | | | 229,420 |
| S24 | S22 NOT S23 | | | 383 |
| S23 | TI ("case report*) | | | 99,307 |
| S22 | S20 OR S21 | | | 385 |
| S21 | S5 AND S6 AND S19 | | | 371 |
| S20 | S5 AND S6 AND S13 | | | 129 |
| S19 | S14 OR S15 OR S16 OR S17 OR S18 | | | 678,394 |
| S18 | MH "Ultrasonography+" | | | 123,508 |
| S17 | MH "Tomography, X-Ray Computed+" | | | 133,509 |
| S16 | MH "Magnetic Resonance Imaging+" | | | 156,800 |
| S15 | MH "Diagnostic Imaging" | | | 36,504 |
| S14 | XB (imag* or (computed N3 tomograph*) or CT or CTs or "magnetic resonance imag*" or MRI or MRIs or ultraso*) | | | 515,844 |
| S13 | S7 OR S8 OR S9 OR S10 OR S11 OR S12 | | | 915,126 |
| S12 | XB (reliability or reliable or reproducibility or reproducible or repeatability or repeatable or agreement or interobserver or intraobserver or inter-observer or intra-observer or interrater or intrarater or inter-rater or intra-rater or kappa or "intraclass correlation" or "intra-class correlation" or validat* or validity) | | | 399,940 |
| S11 | MH "kappa Statistic" | | | 19,541 |
| S10 | MH "Reliability and Validity+" | | | 314,866 |
| S9 | MH "Validation Studies" | | | 357,096 |
| S8 | MH "Observer Bias+" | | | 10,195 |
| S7 | MH "Reproducibility of " | | | 71,579 |
| S6 | XB ("fatty infiltration" or "fatty degeneration" or "intramuscular fat" or "intra-muscular fat" or "muscular fat" or "fat fraction" or "muscle fat" or Goutallier) | | | 2,350 |
| S5 | S1 OR S2 OR S3 OR S4 | | | 11,213 |
| S4 | XB (supraspinat* or supra-spinat* or infraspinat* or infra-spinat* or subscapular* or sub-scapular* or "teres minor*") | | | 3,933 |
| S3 | XB ("rotator cuff*") | | | 7,938 |
| S2 | MH "Rotator Cuff Injuries" | | | 3,766 |
| S1 | MH "Rotator Cuff+" | | | 4,092 |

**4. Web of Science**

Run	27 March 2026

| #1 | "rotator cuff*" or supraspinat* or supra-spinat* or infraspinat* or infra-spinat* or subscapular* or sub-scapular* or "teres minor*" (Topic) | 30,024 |
| --- | --- | --- |
| #2 | "fatty infiltration" or "fatty degeneration" or "intramuscular fat" or "intra-muscular fat" or "muscular fat" or "fat fraction" or "muscle fat" or Goutallier (Topic) | 19,058 |
| #3 | reliability or reliable or reproducibility or reproducible or repeatability or repeatable or agreement or interobserver or intraobserver or inter-observer or intra-observer or interrater or intrarater or inter-rater or intra-rater or kappa or "intraclass correlation" or "intra-class correlation" or validat* or validity (Topic) | 6,377,060 |
| #4 | imag* or (computed NEAR/3 tomograph*) or CT or CTs or "magnetic resonance imag*" or MRI or MRIs or ultraso* (Topic) | 5,605,449 |
| #5 | #1 AND #2 AND #3 | 329 |
| #6 | #1 AND #2 AND #4 | 1,089 |
| #7 | #5 OR #6 | 1,154 |
| #8 | "case report*" (Title) | 398,075 |
| #9 | #7 NOT #8 | 1,149 |
| #10 | #7 NOT #8 and Editorial Material or Review Article or Meeting Abstract (Exclude – Document Types) | 1,064 |

**5. Cochrane Library (CENTRAL)**

Run	27 March 2026

| #1 | MeSH descriptor: [Rotator Cuff] this term only | 705 |
| --- | --- | --- |
| #2 | MeSH descriptor: [Rotator Cuff Injuries] this term only | 1,050 |
| #3 | (rotator NEXT cuff*):ti,ab,kw | 3,482 |
| #4 | (supraspinat* or supra-spinat* or infraspinat* or infra-spinat* or subscapular* or sub-scapular* or (teres NEXT minor*)):ti,ab,kw | 1,477 |
| #5 | #1 OR #2 OR #3 OR #4 | 4,358 |
| #6 | ("fatty infiltration" or "fatty degeneration" or "intramuscular fat" or "intra-muscular fat" or "muscular fat" or "fat fraction" or "muscle fat" or Goutallier):ti,ab,kw | 1,235 |
| #7 | MeSH descriptor: [Reproducibility of Results] explode all trees | 16,876 |
| #8 | MeSH descriptor: [Observer Variation] this term only | 2,700 |
| #9 | MeSH descriptor: [Validation Study] this term only | - |
| #10 | (reliability or reliable or reproducibility or reproducible or repeatability or repeatable or agreement or interobserver or intraobserver or inter-observer or intra-observer or interrater or intrarater or inter-rater or intra-rater or kappa or "intraclass correlation" or "intra-class correlation" or validat* or validity):ti,ab,kw | 125,792 |
| #11 | #7 OR #8 OR #9 OR #10 | 126,702 |
| #12 | (imag* or (computed NEAR/3 tomograph*) or CT or CTs or ("magnetic resonance" NEXT imag*) or MRI or MRIs or ultraso*):ti,ab,kw | 266,201 |
| #13 | MeSH descriptor: [Magnetic Resonance Imaging] explode all trees | 13,752 |
| #14 | MeSH descriptor: [Tomography, X-Ray Computed] explode all trees | 9,151 |
| #15 | MeSH descriptor: [Ultrasonography] explode all trees | 19,855 |
| #16 | MeSH descriptor: [Diagnostic Imaging] this term only | 253 |
| #17 | #12 OR #13 OR #14 O R #15 OR #16 | 266,251 |
| #18 | #5 AND #6 AND #11 | 14 |
| #19 | #5 AND #6 AND #17 | 96 |
| #20 | #18 OR #19 | 97 |
| #21 | #20 NOT (clinicaltrials or trialsearch):so | 60 |
