# Supplementary material for: Reliability of imaging assessment of fatty infiltration in rotator cuff disorders: protocol for a systematic review and meta-analysis
Source: BMJ Open. 2026 Aug 11;16(8):e120564. doi: 10.1136/bmjopen-2026-120564 (PMC13475469; doi:10.1136/bmjopen-2026-120564)
Supplement: Supplementary file 3 [file bmjopen-16-8-s002.docx]

**Supplementary materials 2**

**Operational definitions for QAREL items** **in context of imaging assessment of fatty infiltration in rotator cuff disorders**

The Quality Appraisal of Reliability Studies (QAREL) checklist was used to assess methodological quality ^1^. The checklist comprises 11 items, which are presented in the tables below. Items from the QAREL Data Extraction Form (DEF) were used to inform the rating of each QAREL item. Operational definitions were developed to guide the interpretation of each item within the context of imaging-based assessment of fatty infiltration in rotator cuff disorders.

**Table 1 QAREL Item 1**

| *Was the test evaluated in a sample of subjects representative of the target population?* (DEF: 3, 4, 5, 7, 8, 9) | |
| --- | --- |
| Description | Studies should report:  i) a clearly defined target population and adequate description of participant characteristics (e.g., age, sex, symptomatic status, duration of symptoms, body mass index, rotator cuff status);  ii) the study setting (e.g., primary or tertiary care);  iii) recruitment methods (e.g., consecutive, convenience, or random sampling);  iv) inclusion and exclusion criteria;  v) information on drop-outs or missing data. |
| “Yes” | All of items i)–v) are adequately reported. The target population is clearly defined, and participant characteristics include at least age, sex, and symptomatic status. Study setting, recruitment methods, and eligibility criteria are described, and information on drop-outs or missing data is provided. |
| “No” | None of i)–v) are reported, or the study population is not described. The target population is unclear, and no relevant information on participant characteristics, study setting, recruitment methods, or eligibility criteria is provided. |
| “Unclear” | Reporting is incomplete or insufficient to determine representativeness. For example, the target population is vaguely defined, or key participant characteristics (e.g., symptomatic status) are missing. |

**Table 2 QAREL Item 2**

| *Was the test performed by raters representative of the intended user group?*  (DEF: 3, 4, 6, 7, 8, 9) | |
| --- | --- |
| Description | Studies should report:  i) the intended population of raters;  ii) relevant characteristics of raters, including professional background (e.g., radiologists, orthopaedic surgeons, sonographers), level of experience, and any specific training related to fatty infiltration assessment. |
| “Yes” | The intended population of raters is clearly described, and sufficient information on rater characteristics is provided to allow judgement of representativeness (e.g., professional background and either experience or training). |
| “No” | No information is provided about the raters, or their characteristics are not described. |
| “Unclear” | Reporting is incomplete or insufficient to determine representativeness. For example, only limited information on rater characteristics is provided without sufficient detail on professional background, experience, or training. |

**Table 3 QAREL Item 3**

| *Were raters blinded to the findings of other raters during the study?* (DEF: 10) | |
| --- | --- |
| Description | This item applies only to studies involving more than one rater. Blinding was interpreted as independent assessment without access to the findings of other raters. |
| “Yes” | It is clearly stated that raters performed assessments independently without knowledge of other raters’ findings (e.g., “raters worked independently” or “raters were blinded to other raters’ assessments”). |
| “No” | Raters had access to or were aware of other raters’ findings during the assessment. |
| “Unclear” | The information provided is insufficient to confirm the independence of raters. |
| “Not applicable” | This item was not applicable to studies involving only one rater (e.g., intra-rater reliability assessed by a single rater). |

**Table 4 QAREL Item 4**

| *Were raters blinded to their own prior findings of the test under evaluation?* (DEF: 11) | |
| --- | --- |
| Description | This item applies only to studies evaluating intra-rater reliability. It addresses whether repeated assessments were performed independently, without access to prior ratings, and with appropriate measures to minimise recall bias (e.g., use of separate rating sessions). |
| “Yes” | It is clearly stated that raters performed repeated assessments without access to their prior ratings, and that appropriate steps were taken to minimise recall bias. |
| “No” | Raters had access to their prior ratings, or repeated assessments were conducted under conditions likely to introduce recall bias (e.g., immediate or same-session reassessment). |
| “Unclear” | The information provided is insufficient to determine whether raters were blinded to their prior findings or whether recall bias was minimised. |
| “Not applicable” | This item was not applicable to studies that did not include repeated assessments by the same rater (e.g., inter-rater reliability only). |

**Table 5 QAREL Item 5**

| *Were raters blinded to prior or alternative assessment results of the target variable?* (DEF: 12) | |
| --- | --- |
| Description | No universally accepted reference standard exists for fatty infiltration assessment. Although the Goutallier classification is the most widely used semi-quantitative method, it is not a reference standard. This item was interpreted as whether raters were blinded to prior or alternative assessment results (e.g., previous fatty infiltration grading or measurements) that could influence image interpretation. |
| “Yes” | It is clearly stated that raters were blinded to prior or alternative assessment results. |
| “No” | Raters had access to prior or alternative assessment results that could influence their ratings. |
| “Unclear” | The information provided is insufficient to determine whether raters were blinded to prior or alternative assessment results. |
| “Not applicable” | This item was not applicable if no prior or alternative assessment results were available to the raters. |

**Table 6 QAREL Item 6**

| *Were raters blinded to clinical information that was not intended to form part of the test?* (DEF: 13) | |
| --- | --- |
| Description | This item addresses whether raters had access to clinical or contextual information (e.g., symptoms, diagnosis, treatment history) that was not intended to form part of the imaging-based assessment of fatty infiltration and could influence interpretation. |
| “Yes” | It is clearly stated that raters were blinded to relevant clinical or contextual information not intended to form part of the test. |
| “No” | Raters had access to clinical or contextual information that could influence their ratings. |
| “Unclear” | The information provided is insufficient to determine whether raters were blinded to clinical or contextual information. |

**Table 7 QAREL Item 7**

| *Were raters blinded to additional cues that were not part of the test?* (DEF: 14) | |
| --- | --- |
| Description | This item addresses whether raters were exposed to additional cues not intended to form part of the test and not classified as clinical information, which could influence image interpretation (e.g., visible surgical markers, annotations, or other identifying features in the images). |
| “Yes” | It is clearly stated that raters were blinded to additional cues not intended to form part of the test (e.g., assessment based on anonymised imaging data only). |
| “No” | Raters were exposed to additional cues that could influence their ratings. |
| “Unclear” | The information provided is insufficient to determine whether raters were blinded to additional cues. |

**Table 8 QAREL Item 8**

| *Was the order of examination varied?* (DEF: 15, 16) | |
| --- | --- |
| Description | This item applies to studies involving repeated assessments (e.g., intra-rater reliability). The order in which subjects or images are assessed may introduce order effects and bias. Varying the order of presentation between rating sessions helps to minimise such bias. |
| “Yes” | It is clearly stated that the order of presentation was varied between rating sessions (e.g., randomised order). |
| “No” | The order of presentation was fixed between rating sessions, or no attempt was made to vary the order. |
| “Unclear” | The information provided is insufficient to determine whether the order of presentation was varied. |
| “Not applicable” | This item was not applicable to studies without repeated assessments (e.g., inter-rater reliability conducted in a single session). |

**Table 9 QAREL Item 9**

| *Was the time interval between repeated measurements sufficient to minimise recall bias?* (DEF: 17) | |
| --- | --- |
| Description | This item applies to studies evaluating intra-rater reliability. It assesses whether the time interval between repeated assessments was sufficient to reduce the likelihood that raters could recall their prior ratings when reassessing the same images. |
| “Yes” | The time interval is clearly described and is sufficient to minimise recall bias. |
| “No” | The time interval is clearly described but is too short and likely to introduce recall bias (e.g., immediate or same-session reassessment). |
| “Unclear” | The time interval is not reported, or insufficient information is provided to determine whether it was adequate. |
| “Not applicable” | This item was not applicable to studies without repeated assessments (e.g., inter-rater reliability only). |

**Table 10 QAREL Item 10**

| *Was the test* *applied correctly and interpreted appropriately?* (DEF: 18) | |
| --- | --- |
| Description | The test procedure should be described in sufficient detail to allow understanding of how the assessment was performed. This item considers whether the test was applied appropriately and whether the results were interpreted consistently with the study objectives. |
| “Yes” | Sufficient information is provided to indicate that the test was applied appropriately and interpreted correctly (e.g., use of standardised assessment methods, consistent application of grading or measurement criteria). |
| “No” | The test was applied incorrectly or interpreted inappropriately, or there is clear evidence of inconsistent or inappropriate application of the assessment method. |
| “Unclear” | The information provided is insufficient to determine whether the test was applied and interpreted appropriately. |

**Table 11 QAREL Item 11**

| *Were* *appropriate statistical measures of agreement used?* (DEF: 19, 20, 21) | |
| --- | --- |
| Description | Chance-corrected agreement statistics (e.g., kappa) should be used for categorical data, and ICC for continuous data. Measures of precision (e.g., confidence intervals or other measures of uncertainty) should also be reported. |
| “Yes” | Appropriate statistical methods are used and measures of precision are reported. |
| “No” | Inappropriate statistical methods are used (e.g., correlation coefficients or percentage agreement only). |
| “Unclear” | Appropriate methods are used but measures of precision are not reported. |

**References**

1. Lucas NP, Macaskill P, Irwig L, et al. The development of a quality appraisal tool for studies of diagnostic reliability (QAREL). *J Clin Epidemiol* 2010;63(8):854-61. doi: 10.1016/j.jclinepi.2009.10.002 [published Online First: 2010/01/09]
